# Molecular evidence of *Burkholderia pseudomallei* genotypes based on geographical distribution

**DOI:** 10.7717/peerj.1802

**Published:** 2016-03-15

**Authors:** Noorfatin Jihan Zulkefli, Vanitha Mariappan, Kumutha Malar Vellasamy, Chun Wie Chong, Kwai Lin Thong, Sasheela Ponnampalavanar, Jamuna Vadivelu, Cindy Shuan Ju Teh

**Affiliations:** 1 Department of Medical Microbiology, Universiti Malaya, Kuala Lumpur, Malaysia; 2 Department of Life Sciences, School of Pharmacy, International Medical University, Kuala Lumpur, Malaysia; 3 Institute of Biological Sciences, Universiti Malaya, Kuala Lumpur, Malaysia; 4 Department of Medicine, Universiti Malaya, Kuala Lumpur, Malaysia

**Keywords:** *Burkholderia pseudomallei*, MLST, Central intermediary metabolism, Genetic variants, Geographical distribution

## Abstract

**Background.** Central intermediary metabolism (CIM) in bacteria is defined as a set of metabolic biochemical reactions within a cell, which is essential for the cell to survive in response to environmental perturbations. The genes associated with CIM are commonly found in both pathogenic and non-pathogenic strains. As these genes are involved in vital metabolic processes of bacteria, we explored the efficiency of the genes in genotypic characterization of *Burkholderia pseudomallei* isolates, compared with the established pulsed-field gel electrophoresis (PFGE) and multilocus sequence typing (MLST) schemes.

**Methods.** Nine previously sequenced *B. pseudomallei* isolates from Malaysia were characterized by PFGE, MLST and CIM genes. The isolates were later compared to the other 39 *B. pseudomallei* strains, retrieved from GenBank using both MLST and sequence analysis of CIM genes. UniFrac and hierachical clustering analyses were performed using the results generated by both MLST and sequence analysis of CIM genes.

**Results.** Genetic relatedness of nine Malaysian *B. pseudomallei* isolates and the other 39 strains was investigated. The nine Malaysian isolates were subtyped into six PFGE profiles, four MLST profiles and five sequence types based on CIM genes alignment. All methods demonstrated the clonality of OB and CB as well as CMS and THE. However, PFGE showed less than 70% similarity between a pair of morphology variants, OS and OB. In contrast, OS was identical to the soil isolate, MARAN. To have a better understanding of the genetic diversity of *B. pseudomallei* worldwide, we further aligned the sequences of genes used in MLST and genes associated with CIM for the nine Malaysian isolates and 39 *B. pseudomallei* strains from NCBI database. Overall, based on the CIM genes, the strains were subtyped into 33 profiles where majority of the strains from Asian countries were clustered together. On the other hand, MLST resolved the isolates into 31 profiles which formed three clusters. Hierarchical clustering using UniFrac distance suggested that the isolates from Australia were genetically distinct from the Asian isolates. Nevertheless, statistical significant differences were detected between isolates from Malaysia, Thailand and Australia.

**Discussion.** Overall, PFGE showed higher discriminative power in clustering the nine Malaysian *B. pseudomallei* isolates and indicated its suitability for localized epidemiological study. Compared to MLST, CIM genes showed higher resolution in distinguishing those non-related strains and better clustering of strains from different geographical regions. A closer genetic relatedness of Malaysian isolates with all Asian strains in comparison to Australian strains was observed. This finding was supported by UniFrac analysis which resulted in geographical segregation between Australia and the Asian countries.

## Introduction

*Burkholderia pseudomallei* is an environmental saprophytic Gram-negative bacterium that causes melioidosis, a potentially fatal bacterial infection. Infection can be acquired through percutanous inoculation, inhalation or ingestion of either contaminated soil or water (Wiersinga, Currie & Peacock, 2012). Southeast Asia and northern Australia are listed as predominantly endemic for melioidosis (White, 2003; Puthucheary, 2009). Sporadic cases have also been reported in non-endemic regions such as Africa, India and United States (Wiersinga, Currie & Peacock, 2012). The clinical presentations of this disease vary largely among patients, but the most common are pneumonia and septicaemia (Cheng & Currie, 2005). Certain clinical presentations may display region-specific predominance. For instance, genitourinary infections are more prevalent in Australia while hepatosplenic abscesses occur more frequently in Thailand (Cheng & Currie, 2005; Limmathurotsakul & Peacock, 2011). However, to date, no correlation has been reported between the clinical presentations and genotypes, although geographical partitioning between Australian and Asian population of *B. pseudomallei* has previously been reported (Cheng et al., 2004; Koonpaew et al., 2000)

Analyses on the relatedness among bacterial strains using genotyping methods aid to determine source of outbreak or infection (local epidemiology) and understand the diversity and evolution of a microbial population (global epidemiology) (Carrico et al., 2013). Genotyping mainly distinguishes bacterial strains based on genetic variation resulted from mutation or recombination, and selection pressure on the genome (Urwin & Maiden, 2003). The plethora of genotyping methods can be grouped into two categories; DNA pattern typing and DNA sequence typing (Carrico et al., 2013). Pulsed-field gel electrophoresis (PFGE) is an example of DNA pattern typing method, where bacterial genome is cleaved by restriction enzymes and divided into different lengths of DNA fragments. The DNA fragments are subsequently separated on an agarose gel, which form banding patterns known as pulsotype (Sabat et al., 2013). This method is comparatively inexpensive, highly discriminative and has excellent typeability, but it is tedious, time-consuming and limited in portability (Sabat et al., 2013). PFGE has been extensively used in subtyping of *B. pseudomallei* strains in Malaysia and high level of diversities was observed. However, based on PFGE, the strains from East Malaysia could not be differentiated from those isolated in Peninsular Malaysia (Chua et al., 2010; Chua et al., 2011).

On the other hand, DNA amplification-based typing such as multilocus sequence typing (MLST) utilizes nucleotide sequences to differentiate strains (Maiden et al., 1998). The unambiguous nature of nucleotide sequences allows interlaboratory comparisons to be performed and thus overcome the portability issue of other molecular typings (Maiden et al., 1998; Sabat et al., 2013). The MLST scheme utilizes seven housekeeping genes that are sequenced, combined and assigned to a sequence type (ST) according to the allelic profiles deposited on MLST.net (http://bpseudomallei.mlst.net/) (Godoy et al., 2003). Housekeeping genes are selected as gene markers in MLST since genetic variation accumulates slowly for metabolic function, thus making it useful for long-term epidemiology monitoring (Maiden et al., 1998; Urwin & Maiden, 2003). Assuming that gene mutations of the housekeeping genes are under stabilizing selection, then the clonality of the species is conserved (Maiden et al., 1998; Urwin & Maiden, 2003). However, it is not suitable for local surveillance of some pathogens as it is insufficiently discriminative (Urwin & Maiden, 2003; Sabat et al., 2013). Modifications to the MLST schemes have been proposed to improve the discriminatory or resolution power of the “conventional” MLST scheme. The MLST schemes of *Neisseria meningitidis* and *Salmonella enterica* demonstrated better discriminatory power with the addition of *fumC* gene, and a combination of two housekeeping (*gyrB* and *atpD*) and flagellin (*fliC* and *fliB*) genes, respectively (Holmes, Urwin & Maiden, 1999; Tankouo-Sandjong et al., 2007).

Central intermediary metabolism (CIM) in bacteria is important as it plays an essential role for the cell to grow, survive and response to environmental perturbations. In living cells, thousands of different biochemical reactions and transport processes are linked together to generate constant supply of energy and to maintain their biological functions (Spector, 2009; Shimizu, 2013). The genes associated with CIM are housekeeping genes that encode metabolic functions, hence CIM genes are considered good molecular markers for the study of phylogenetic relationship at species level (Naharro, Rubio & Luengo, 2011). In this study, we tested the feasibility of the CIM genes as alternative approach for strains characterization. The genetic relatedness of the strains that were previously deposited in the National Center for Biotechnology Information (NCBI) Genbank (http://www.ncbi.nlm.nih.gov/genome/476) was determined based on MLST and the genes related to CIM. This study may provide better insight into the genetic diversity of *B. pseudomallei* and epidemiological distribution of *B. pseudomallei* population in Malaysia and in comparison to other countries.

## Methods

### Genomic sequences and bacterial isolates

Nineteen complete and 29 draft genomes of *B. pseudomallei* were selected from NCBI Genbank (http://www.ncbi.nlm.nih.gov/genbank/) based upon the availability of background information (e.g., source, year of isolation, country) in order to determine geographical relatedness. Among the 48 genomes selected, nine of them were from Malaysia (Table 1).

**Table 1:** Background information and MLST analysis of *B. pseudomallei* genomes used in this study.

Accession number	Strain	Source	Country	Year	ST	Allele profile						
						*ace*	*gltB*	*gmhD*	*lepA*	*lipA*	*narK*	*ndh*
AGVS01	354a	Sputum	Thailand	1988	78	1	2	2	1	1	1	1
AHJD01	354e^a^	Sputum	Thailand	1994	78	1	2	2	1	1	1	1
AAMM02	406e	Toe swab	Thailand	1988	211	3	1	3	1	1	4	1
NZCP008777	576	Human	Thailand	1989	501	1	12	6	2	1	2	3
AHJA01	1026a	Pus	Thailand	1993	102	3	4	12	1	1	4	1
NC017851	1026b	Blood	Thailand	1993	102	3	4	12	1	1	4	1
AAMB02	1106b	Pus	Thailand	1996	70	3	4	11	3	5	4	6
AHJB01	1258a	Sputum	Thailand	1994	221	1	12	2	3	5	29	1
AHJC01	1258b^b^	Blood	Thailand	1995	221	1	12	2	3	5	29	1
ABBN01	B7210	Pus	SE Asia	–	84	3	1	11	4	5	4	6
JNOW01	BDD	Blood	Australia	–	956	1	2	13	2	1	45	11
JPNW01	BDE	Sputum	Australia	–	140	4	7	3	4	1	19	1
JPNU01	BDI	Blood	Australia	–	252	1	4	3	2	6	19	1
NZCP009209	BDP	Brain	Australia	1994	36	1	7	14	7	1	12	11
JOTS01	BDT	Joint aspirate	Australia	–	958	1	2	14	22	1	8	1
JPNP01	BEB	Blood	Australia	–	254	1	4	3	2	2	34	1
JPNQ01	BED	Blood	Australia	–	24	1	2	13	2	1	8	1
	BEJ	–	Thailand	–	15	1	2	2	2	1	3	1
	BEM	–	Vietnam	–	411	1	4	4	3	1	3	1
	BEX	–	Thailand	–	501	1	12	6	2	1	2	3
	BFD	Blood	Australia	–	445	8	4	13	2	1	8	1
	BGR	–	Thailand	–	102	3	4	12	1	1	4	1
NC018527	BPC006	Blood	China	2008	70	3	4	11	3	5	4	6
APLM01	CB	Blood	Malaysia	–	46	3	1	2	1	1	3	3
APLH01	CMS	Blood	Malaysia	–	54	3	1	3	3	1	2	1
APLN01	CS^c^	Blood	Malaysia	–	46	3	1	2	1	1	3	3
AVAL01	HBPUB10134a	Sputum	Thailand	2010	228	1	2	3	1	1	4	1
AVAM01	HBPUB10303a	Sputum	Thailand	2011	48	3	1	2	1	1	4	1
APLG01	LMF	Sputum	Malaysia	–	289	3	4	11	4	5	4	6
NZCP008781	Mahidol-1106a	–	Thailand	–	70	3	4	11	3	5	4	6
APHN01	MARAN	Soil	Malaysia	–	1342	4	12	3	2	1	1	3
NZCP004042	MSHR146	Goat	Australia	1992	617	1	16	3	4	2	8	1
NC021877	MSHR305	Brain	Australia	1994	36	1	7	14	7	1	12	11
NZCP008764	MSHR346	Sputum	Australia	1995	243	1	2	13	4	15	12	1
NZCP004023	MSHR511	Goat	Australia	1997	617	1	16	3	4	2	8	1
NZCP004368	MSHR520	Blood	Australia	1998	36	1	7	14	7	1	12	11
AVAJ01	MSHR5848	Sputum	Australia	2011	553	1	1	3	4	30	2	30
JMMV01	MSHR5855	Sputum	Australia	2011	553	1	1	3	4	30	2	30
AVAK01	MSHR5858	Sputum	Australia	2011	562	1	1	4	1	1	29	1
AXDS01	MSHR6137	Water	Australia	2012	325	1	1	13	4	15	3	11
NZCP004003	NAU20B-16	Soil	Australia	2006	617	1	16	3	4	2	8	1
NZCP004001	NCTC 13178	Brain	Australia	–	286	15	2	13	2	20	19	1
NC022659	NCTC 13179	Skin ulcer	Australia	–	613	1	4	43	4	1	48	11
APLK01	OB	Blood	Malaysia	–	46	3	1	2	1	1	3	3
APLL01	OS^d^	Blood	Malaysia	–	46	3	1	2	1	1	3	3
ACKA01	Pakistan 9	Human	Pakistan	1988	72	4	1	4	1	1	2	1
APLJ01	THE	Spleen	Malaysia	–	54	3	1	3	3	1	2	1
APLI01	VEL	Sputum	Malaysia	–	46	3	1	2	1	1	3	3

**Notes.**

a 354e is a relapse case of 354a.

b 1258b is a relapse case of 1258a.

c CS is the small colony variant of CB.

d OS is the small colony variant of OB.

### Pulsed-field gel electrophoresis

The nine Malaysian isolates were characterized by PFGE analysis. PFGE preparation was performed with a few modifications based on the CDC One-Day Standard Laboratory Protocol for Molecular Subtyping (Ribot et al., 2006). In brief, *B. pseudomallei* isolates were grown overnight on nutrient agar plates at 37 °C. The bacterial cells were suspended in normal saline solution and adjusted to a concentration of OD_600_ = 0.6–0.8 using Dade Behring Microscan Turbidity Meter (Siemens, Germany). The suspensions were mixed with 1.0% (w/v) agarose gel (Seakem Gold Agarose, Rockland, ME, USA) to prepare plugs. The plugs were lysed overnight at 50 °C before they were digested with *SpeI* (Promega, Wisconsin, USA) for an additional 2 h (50 °C). The digested DNA fragments were then loaded into a 1.0% (w/v) agarose gel and separated by electrophoresis using the CHEF Mapper apparatus (Bio-Rad Laboratories, Hercules, CA, USA) as previously described (Chua et al., 2011). The gel was visualized under ultraviolet light using the Gel Doc 2000 system (Bio-Rad Laboratories, Hercules, CA, USA). Analysis was performed using the Bionumerics 6.0 software (Applied Maths, Sint-Martens-Latem, Belgium) to construct a Dice coefficient dendrogram based on the unweighted pair group method with arithmetics average (UPGMA) algorithm.

### Multilocus sequence typing (MLST)

*In silico* MLST analyses were performed by aligning *B. pseudomallei* genomes against the sequences of seven alleles of *B. pseudomallei* K96243 (accession number BX571965) retrieved from *B. pseudomallei* MLST database (http://bpseudomallei.mlst.net/) using basic local alignment search tool (BLASTN) (https://blast.ncbi.nlm.nih.gov/).The alignment was downloaded and the sequence for each allele (gene) was submitted to the *B. pseudomallei* MLST database to assign the allelic numbers (Godoy et al., 2003). A combination of the seven allelic numbers by the order of *ace-gltB-gmhD-lepA-lipA-narK-ndh* was compared against the *B. pseudomallei* MLST database to obtain a ST number (Godoy et al., 2003).

### Genetic characterization based on central intermediary metabolism (CIM) genes

Seventy CIM gene sequences of K96243 (accession number BX571965) were obtained from the *Burkholderia* Genome Database (http://beta.burkholderia.com/)(Holden et al., 2004; Winsor et al., 2008). *B. pseudomallei* genomes were aligned against the CIM gene sequences using basic local alignment search tool (BLASTN) (https://blast.ncbi.nlm.nih.gov/). Twelve CIM genes were finally selected as only these genes showed 100% query coverage across all 48 strains.

### Maximum likelihood analysis

Phylogenetic trees for the nine isolates from Malaysia and 48 strains worldwide were constructed using the concatenated sequence of MLST housekeeping genes and CIM genes respectively, based on the maximum likelihood method. The seven genes of MLST and 12 CIM genes were concatenated in order of *ace-gltB-gmhD-lepA-lipA-narK-ndh* and BPSL0512-BPSL0958–BPSL1410–BPSL1418–BPSL1668–BPSL2657–BPSL2658–BPSL2659–BPSL2662–BPSL2837–BPSS0626–BPSS1635, respectively. The trees were constructed using MEGA6.06 software (Molecular Evolutionary Genetics Analysis software, Arizona State University, Tempe, AZ, USA) with 1,000 bootstrap replications (Tamura & Nei, 1993; Tamura et al., 2013).

### Discriminatory index

The discriminatory index (*D*) of MLST and sequence analysis of CIM genes were calculated using the formula of Simpson’s index of diversity (Hunter & Gaston, 1988). An index of 1.0 indicated that the typing method was able to differentiate each member of a strain population as unique. On the other hand, *D* value of 0 indicated that all members of a strain population were identical.

### Comparison of CIM, MLST and WGS

Phylogenetic relationship of 15 randomly selected *B. pseudomallei* genomes was established based on Tetra-nucleotide signature correlation index calculated from JSpecies Web Server (http://jspecies.ribohost.com/jspeciesws) (Richter et al., 2015). The server allowed a maximum of fifteen genomes to be compared per session. The correlation matrix was matched with the maximum likelihood distance matrices produced from the corresponding concatenated CIM and MLST genes using Spearman Rank Correlation (*ρ*). Significant correlation was calculated based on 1,000 permutations.

### Unweighted UniFrac analysis

Unweighted Unifrac analysis (Lozupone, Hamady & Knight, 2006) was conducted using Mothur (ver 1.36.1) (Schloss et al., 2009) to identify significant differences in geographical distribution. In brief, the distance matrix (shared distance between strains) was calculated based on individual maximum likelihood trees generated from MLST and CIM genes analyses. The significance was calculated through Monte Carlo permutation with 1000 times resampling. The relationship between these geographical locations was illustrated using hierarchical clustering under Ward’s method. The calculation was conducted using “hcluster” command under R programme.

## Results

### Selection and diversity of central intermediary metabolism genes

A total of 12 CIM genes (100% coverage) were selected for the analysis and these genes were present in all the 48 *B. pseudomallei* genomes selected from the NCBI GenBank database (Table 2). The ratio of polymorphic sites in the CIM genes ranged from 0.9% to 2.5%. (Table 2)

**Table 2:** Properties of the 12 selected CIM genes.

Locus tag	Gene name	Product name	Size for analysis (bp)	No. of alleles	No of variable sites (%)	Genome location (kb)
BPSL 0512	–	Nitrite reductase	2445	25	34 (1.4)	564
BPSL 0958	*cysH*	Phosphoadenosine phosphosulfate reductase	750	15	15 (2.0)	1,116
BPSL 1410	–	Peptidyl-prolyl cis-trans isomerase	1935	22	30 (1.6)	1,640
BPSL 1418	–	Exported isomerase	780	12	13 (1.7)	1,654
BPSL 1668	–	Adenylylsulfate kinase	852	18	21 (2.5)	1,946
BPSL 2657	*ureA*	Urease subunit gamma	303	5	4 (1.3)	3,181
BPSL 2658	*ureB*	Urease subunit beta	306	9	7 (2.3)	3,181
BPSL 2659	*ureC*	Urease subunit alpha	1707	27	34 (2.0)	3,182
BPSL 2662	*ureG*	Urease accessory protein	651	14	12 (1.8)	3,184
BPSL 2837	–	Carbon-nitrogen hydrolase	828	13	10 (1.2)	3,396
BPSS 0626	–	Luciferase-like monooxygenase	1095	9	10 (0.9)	854
BPSS 1635	–	Class III aminotransferase	2787	26	60 (2.2)	2,248

### Relationship between nine Malaysian *B. pseudomallei* isolates

**Figure 1:**
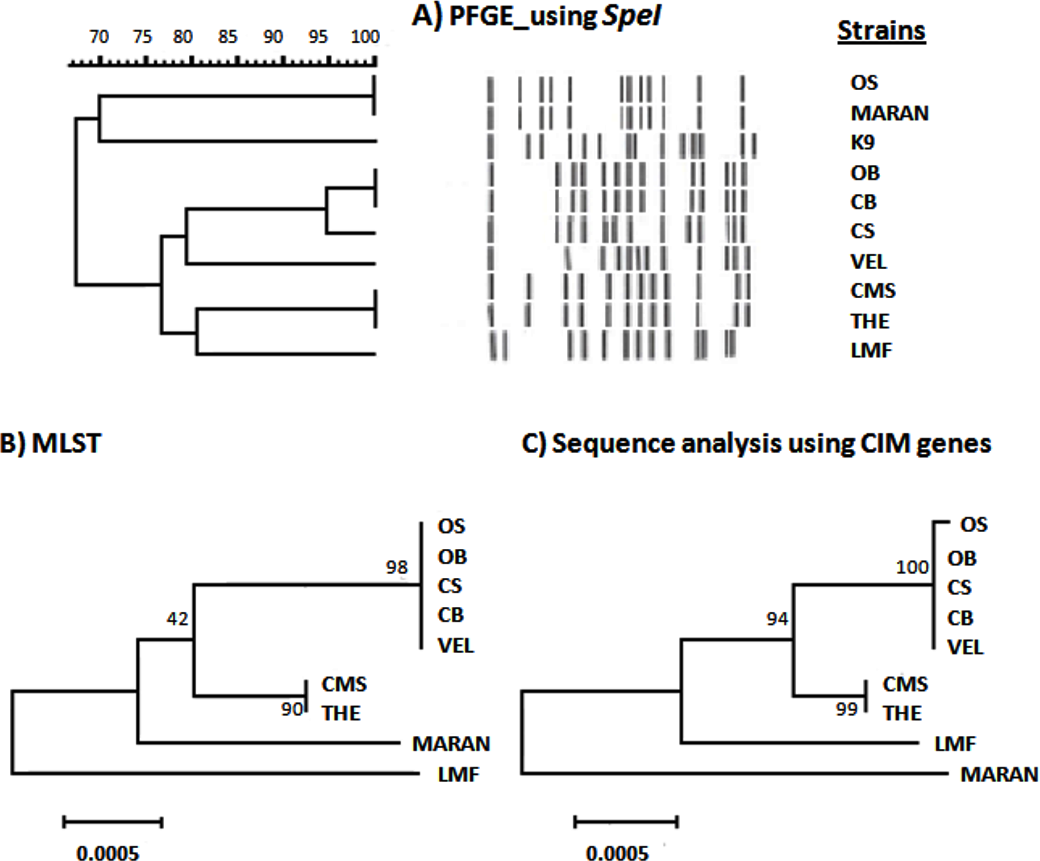
Comparison between PFGE, CIM genes and MLST profiles of nine *Burkholderia pseudomallei* isolates from Malaysia. (A) Dendrogram generated using UPGMA based on Dice coefficients showed two clusters at 65% similarity. K9 was the *B. pseudomallei* reference strain, K96243. (B) Maximum likelihood tree generated using 7 MLST genes showed the close relationship of the colony variants (OB, OS; CB, CS) with the other clinical isolates and LMF and MARAN as outgroups. (C) The relationship of the Malaysian isolates were also depicted using maximum likelihood tree based on sequence analysis of CIM genes. The tree showed the slight difference of OS with its big colony variant, OB. The values on the nodes indicate the bootstrap value with 1,000 times resampling for (B) and (C).

The relatedness of nine Malaysian *B. pseudomallei* strains was determined based on PFGE, MLST and sequence analysis of CIM genes (Fig. 1). Based on PFGE analysis (Fig. 1A), these nine Malaysian strains were resolved into six pulsotypes. Two clusters were observed at 75% similarity with the exclusion of the *B. pseudomallei* K96243. Interestingly, the similarity level between morphology variants, OS/OB and CS/CB was only 67%, and 95%, respectively. In contrast, two strains, OS (clinical strain) and MARAN (soil strain) have an identical pulsotype despite being isolated from different sources. Both of the isolates were found to be distinct from the rest of the strains. In addition, several other isolates were also sharing identical pulsotypes, namely, CB and OB; CMS and THE. On the other hand, four profiles were observed based on MLST analysis and five profiles were generated based on the CIM genes. However, the phylogenetic trees generated by both sequence-based analyses showed high similarity of clustering pattern, where seven isolates were grouped into one cluster while MARAN and LMF were categorized as outgroups. The morphology variant, OS and OB, which shared similar ST were distinguished based on a single nucleotide polymorphism (SNP) observed in one of the CIM genes, BPSL 0512.

### Relationship between *B. pseudomallei* isolates in Malaysia and other countries

**Figure 2:**
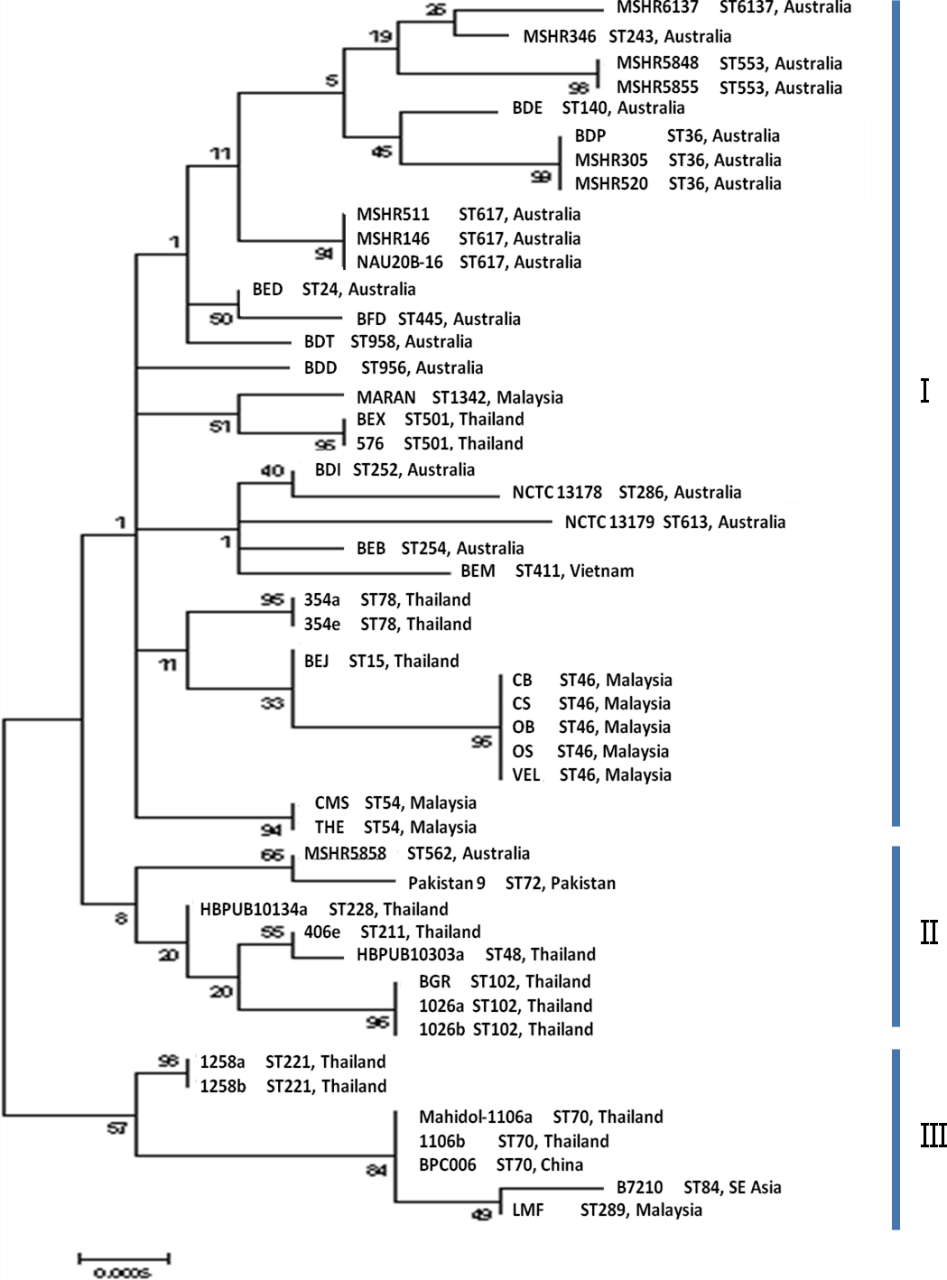
Maximum likelihood tree of MLST displaying the relatedness of the 48 *B. pseudomallei* isolates from endemic countries. The sequence types and countries were indicated for comparison. Three clusters were identified and labeled as cluster I, II and III. The values on the nodes indicate the bootstrap value with 1,000 times resampling.

MLST analysis revealed a novel allelic profile of 4-12-3-2-1-1-3 for MARAN and was assigned with ST1342. Based on the maximum likelihood tree of the seven housekeeping genes (Fig. 2), the 48 *B. pseudomallei* genomes were resolved into 31 profiles and divided into three main clusters (cluster I, II and III). Cluster II and III were mostly comprised by strains from Asian countries, except for MSHR5858 (Australia); while Cluster I was comprised of a mixture of Australian and Asian strains. The eight Malaysian strains were observed in cluster I and majority of the strains were more related to strains from Thailand. Only one strain, LMF was observed in cluster III and was also related to strains from Thailand, China and Southeast Asia. .

**Figure 3:**
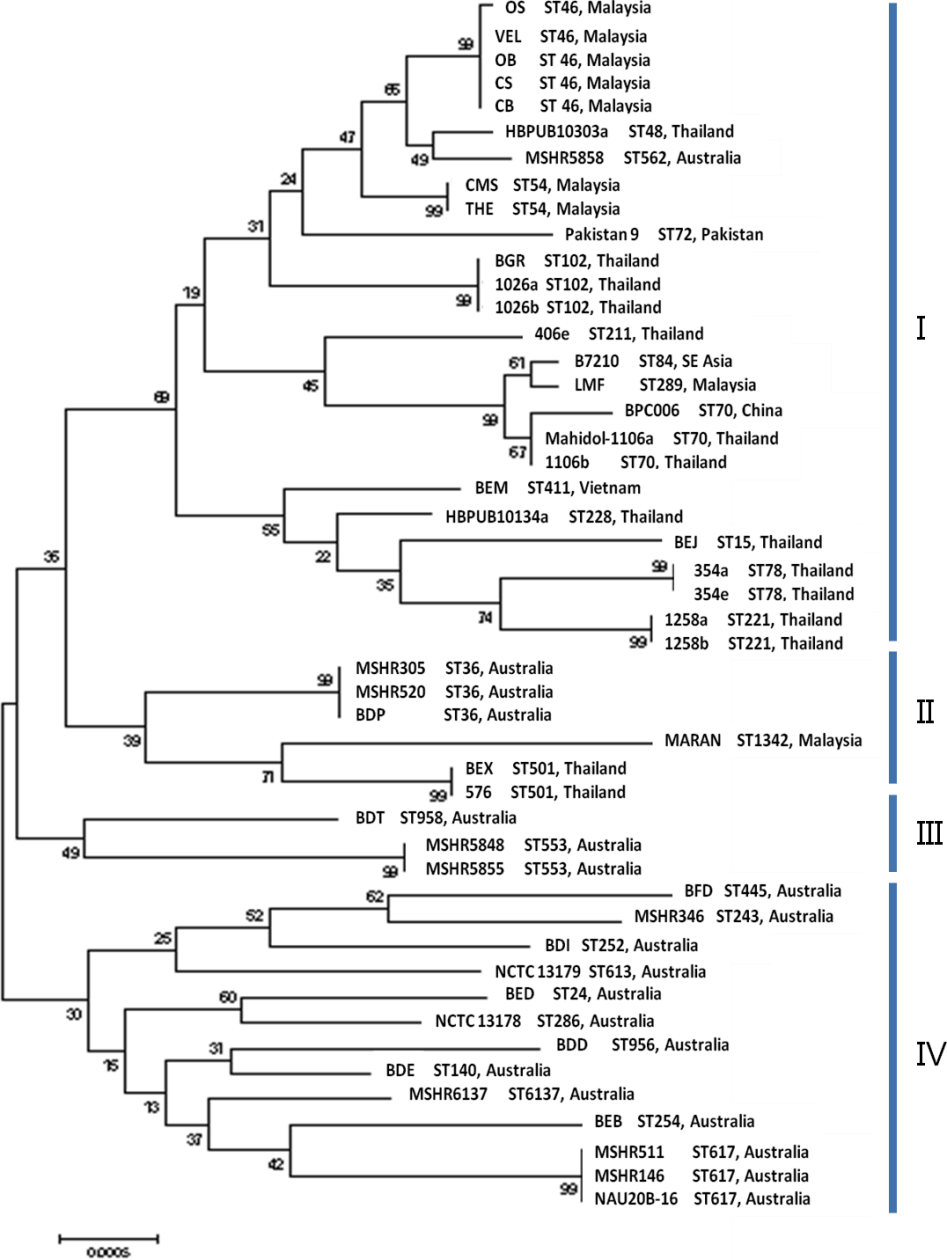
Maximum likelihood tree of sequence analysis of CIM genes displaying the relatedness of the 48 *B. pseudomallei* isolates from endemic countries. The sequence types and countries were indicated for comparison. Cluster A is divided into two subclusters (AI and AII) and mostly occupied by isolates from Asia. Cluster B and C comprised of mostly isolates from Australia. The values on the nodes indicate the bootstrap value with 1000 times resampling.

Based on the maximum likelihood tree generated using CIM genes (Fig. 3), the 48 strains were grouped into 33 profiles and four clusters (I, II, III and IV). All strains in cluster I were from the Asian countries and all the strains with identical profiles were also found to be of the same STs. Among the nine Malaysian strains, MARAN was the most distinctive as it was clustered in cluster II with three strains from Australia (MSHR305, MSHR520 and BDP) and 2 strains from Thailand (BEX and 576). Strains isolated from Australia showed higher diversity as they were distributed across cluster II, III and IV. Overall, discriminatory (D) index of sequence analysis of CIM genes (*D* = 0.9814) is slightly higher than MLST (*D* = 0.9761).

### Comparing the phylogeny inferred from CIM and MLST with WGS

Pairwise genetic distance of fifteen randomly selected isolates inferred from both CIM and MLST was compared with tetra correlation index of WGS using Spearman Rank Correlation. A moderate but significant correlation was established between CIM and MLST (*ρ* = 0.42, *P* = 0.002). However, distance matrices of both CIM and MLST showed no correlation to the phylogeny established with WGS (*ρ* = − 0.06–0.04, *P* > 0.25).

### Geographical relationship among *B. pseudomallei* variants

**Figure 4:**
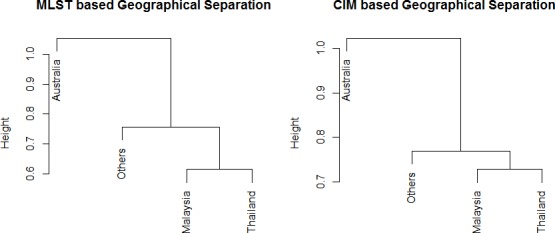
Single linkage hierachical clustering generated based on Unifrac distances of (A) MLST and (B) CIM genes to associate the relatedness according to countries. Genetical distances of *B. pseudomallei* isolates inferred from both MLST and CIM produced similar “geographical structure.” However, CIM exhibited an overall higher resolution in discerning isolates from different region in comparison to MLST.

UniFrac analysis was performed to determine the geographical relationship between the studied strains. Based on both MLST and sequence analysis of CIM genes, significant (*P* ≤ 0.01) geographical clustering was observed (Table 3). For instance, *B. pseudomallei* strains from Australia were found to be distinct from the strains obtained from the Asian countries (*P* ≤ 0.05). Single linkage hierarchical clustering based on Unifrac distances of MLST and CIM analyses produced similar “geographical structure.” However, CIM exhibited an overall higher resolution in discerning isolates from different region in comparison to MLST (Fig. 4). For instance, the distance between Malaysian and Thailand strains is approximately 0.6 in MLST but >0.7 in CIM.

**Table 3:** Pairwise comparison of UniFrac distances based on (A) CIMT and (B) MLST. Statistical significance was calculated using Monte Carlo permutation with 1,000 iterations. Signifance results (*P* ≤ 0.05) are red-color-coded.

A	Australia	Malaysia	Others	Thailand
Australia		0.002	0.103	<0.001
Malaysia			0.204	0.014
Others				0.173
Thailand				

B	Australia	Malaysia	Others	Thailand
Australia		<0.001	0.116	<0.001
Malaysia			0.479	0.044
Others				0.318
Thailand				

## Discussions

CIM is important in bacteria as it plays an essential role for the cell to grow, survive and response to environment perturbation. The genes associated with CIM are commonly found in both pathogenic and non-pathogenic strains. The genes are under stabilizing selection and usually species-specific (Spector, 2009). In this study, a higher *D* index for the alignment of CIM gene was observed compared to MLST. The slightly higher *D* index of the sequence analysis of CIM genes is not significant due to the small sample size (*N* < 100) and might be attributed to the use of 12 full-length genes (303–2787 bp) as opposed to the 7 internal fragments of housekeeping genes in MLST (300–500 bp). Increased numbers of sequence lengths have shown an increase in discriminatory power (Maiden et al., 1998; Godoy et al., 2003; Urwin & Maiden, 2003). Nonetheless, the finding in this study may facilitate further study to assess the variable regions within each CIM sequence and translate them into viable genotyping method. Although WGS is gradually being used as a tool for strains identification and characterization, it is still unlikely to be cost effective for developing countries. Moreover, the laboratory personnel must acquire skills to handle new generation sequencing technology and interpret cumbersome WGS data (Aliyu, 2014).

A direct comparison of the three typing methods for characterization of the Malaysian strains showed that the gel-based PFGE method was more discriminative compared to the other two sequence-based methods (MLST and sequence analysis of CIM genes). This finding concurred with previous studies that reported different PFGE banding patterns can be observed from a single ST (Godoy et al., 2003; Cheng et al., 2004; Chantratita et al., 2008). In general, MLST and CIM genes analyses involved essential metabolic genes that are highly conserved. Thus, these methods are unaffected by genomic rearrangement, whereas PFGE that cleave bacterial genome at restriction sites would produce a different banding pattern if there is any large insertion or deletion along the genomic sequence (Chantratita et al., 2008; Cooper & Feil, 2004). Another possible reason would be the longer history of mutation accumulation and history of lateral gene transfer in *B. pseudomallei*, which leads to homoplasy and apparent homoplasy, respectively, in unrelated isolates (Pearson et al., 2009). For instance, substantial diversity within strains from Australia and Cambodia that shared same STs due to homoplasy had been reported by De Smet et al. (2015). This also indicates the limitation of MLST for characterizing highly recombinogenic species. Overall, our study suggested that PFGE is more suitable for localized epidemiology as it is affected by genomic rearrangements while the sequence-based analyses are more suitable for cross geographical or long-term epidemiological study as previously indicated by Maiden et al. (1998).

In this study, a novel allelic profile (ST1342) was assigned to MARAN, the only soil isolate from Malaysia. Interestingly, PFGE showed that this strain was identical to OS, a clinical isolate and the morphology variant of OB. According to MLST analysis, OS was identified as ST46 and was genetically similar to the other strains of ST46 such as OB, CB, CS and VEL. However, CIM genes analysis revealed a SNP different between OS and other strains of ST46 and indicated that there was a lack of relatedness between OS and the morphology variant, OB as demonstrated by PFGE. To investigate the genetic relatedness of OS, OB and MARAN, further analysis such as WGS will be performed.

On the other hand, both MLST and CIM genes analyses managed to identify the isolates associated with two relapse cases (354a & 354e; 1258a & 1258b), despite the 330 Kb deletion on chromosome 2 of 1258a and 800 Kb inversion on chromosome 2 of 354e that were previously reported by Hayden et al. (2012). In this case, MLST was not affected by the mutations mostly because all the MLST loci are located along chromosome 1. Sequence analysis comprised of two CIM genes on chromosome 2 (BPSS0626 and BPSS1635) yet the two pair isolates were resolved as clonal.

To determine whether the CIM markers are better at recapitulating the whole genome phylogeny compared to the MLST markers, the phylogenetic relationship of 15 randomly selected *B. pseudomallei* genomes has been determined using the online server. Based on the 15 selected *B. pseudomallei* strains, distance matrices of both CIM and MLST showed no correlation to the phylogeny established with WGS but a significant correlation was established between CIM and MLST. Overall, CIM genes analysis showed higher resolution in depicting the relationship of the related and non-related strains compared to MLST. For instance, MLST identified two strains from Thailand and one strain from China as ST46; however CIM genes analysis managed to distinguish the strain from China from the other two strains from Thailand. Based on MLST analysis, the strains from Australia and Asian countries are grouped in one big cluster but clustering analysis using CIM genes showed that the strains from Asian were genetically related as they were clustered in cluster I, whereas the strains from Australia were more diversified as they were distributed across clusters II, III and IV (Cheng et al., 2008; Pearson et al., 2009).

UniFrac analysis revealed strong geographical segregation between strains from Australia and the Asian countries from both sequence-based analyses. This finding concurred with previous studies which reported that STs from Australia were distinctly different from those from Southeast Asian countries (Cheng et al., 2004; Chantratita et al., 2008). Such a distribution might be due to the geographical isolation of Australia from the continent of Asia (Torpdahl et al., 2005; Hayden et al., 2012). Nevertheless, a few STs were found in both Australia and Southeast Asia since the increased number of human migrations between the two regions has been observed (McCombie, Finkelstein & Woods, 2006). Melioidosis cases have been reported on military personnel and travelers who traveled to endemic countries such as Thailand, Vietnam and Australia (Limmathurotsakul & Peacock, 2011; Wiersinga, Currie & Peacock, 2012). The migration of animals across a land bridge connecting Australia and Southeast Asia during the Miocene period has also resulted in common genotype shared by both regions (Cheng et al., 2004).

In addition, extensive studies have been conducted to support the phylogeographic partitioning between Australian and Asian *B. pseudomallei* isolates. Pearson et al. (2009) postulated that *B. pseudomallei* was introduced to Southeast Asia following collision of these two continents in the vicinity of Wallace’s Line after the late Miocene period. Another possible dispersal route is through Papua New Guinea since the island is located between the two endemic regions of Northern Australia and Southeast Asia and the strains from the island showed shared characteristics of both regions (Baker et al., 2011). Based on the study of Tuanyok et al. (2007), Australian and Asian *B. pseudomallei* populations can be genetically differentiated by a *Yersinia*-like fimbrial (YLF) gene cluster, predominantly found in the Asian population, while a *B. thailandensis*-like flagellum and chemotaxis (BTFC) gene cluster is mainly found in Australian population. However, comparison based on SNPs from WGS inferred that the isolates from the two continents are not separated as a result of uneven geographic sampling. Nevertheless, homoplasy should be taken into consideration when phylogenetic relatedness of *B. pseudomallei* strains from different regions is being studied based on MLST as it could be due to recombination and lateral gene transfer that resulted in similar characteristics of unrelated samples (Pearson et al., 2009).

## Conclusions

Genetic relatedness of strains isolated from Malaysia and other countries have been determined and a significant geographical segregation of *B. pseudomallei* genomes between Australia and Asian countries were observed. Overall, the sequence analysis of CIM genes is comparable to MLST and may be useful to be developed into genetic markers.

## Additional Information and Declarations

### DNA Deposition

The following information was supplied regarding the deposition of DNA sequences:
http://bpseudomallei.mlst.net/.

### Data Availability

The following information was supplied regarding data availability: The research in this article did not generate any raw data.
